# Editorial: The role of monoamine oxidase inhibition in smokers: Toward understanding their potential effects in reinforcing nicotine dependence

**DOI:** 10.3389/fnins.2022.1094549

**Published:** 2022-11-21

**Authors:** Penelope Truman

**Affiliations:** School of Health Sciences, Massey University, Wellington, New Zealand

**Keywords:** monoamine oxidase inhibition, nicotine, tobacco dependence, mental health, tobacco smoke constituents, public health

This special issue was proposed with the aim of bringing into focus an area of knowledge deficit within tobacco dependence, that of the role monoamine oxidase (MAO) inhibition may (or may not) play in establishing or reinforcing nicotine dependence.

We obtained five contributions.

Our own Hong et al. provided a brief overview of what is currently known about the pharmacologically active (and potentially beneficial) components of tobacco smoke. The overall conclusion was that nicotine and its metabolite cotinine may well have positive effects on cognitive processes in adults. An array of plant metabolites (identified from other plants and which are used medicinally) are present in tobacco and tobacco smoke but are of unknown significance. The evidence to date suggests that further monoamine oxidase inhibitors in tobacco smoke remain to be identified.

Two contributions directly addressed the effect of monoamine oxidase inhibition on nicotine dependence, providing interesting insights into the effects these enzyme inhibitors may have on behavior.

The paper by Harris et al. reported the differential effects of nicotine, a tobacco smoke extract, and an e-cigarette extract, matched for nicotine concentration, on intracranial self-stimulation (ICSS). They found a clear difference in ICSS responses between cigarette smoke extracts and nicotine alone, with cigarette smoke extracts increasing the threshold for responding, indicating a reduced abuse liability, contrary to initial expectations. E-cigarette vapor extract was not significantly different from nicotine alone in the behavioral responses measured, nor did it contain significant MAO inhibitory activity. The tobacco smoke extract did contain MAO inhibitory activity but the extent of this did not significantly affect brain MAO activity. The observed change in behavioral response supports the contention that MAO inhibitors, or other components of tobacco smoke, affect responses to nicotine. These results did not, however, suggest that components of tobacco smoke other than nicotine are responsible for the high abuse liability of smoked tobacco, instead acting to increase the aversive effects of nicotine in this experimental system. The authors suggest that more studies, using a wider variety of experimental approaches are needed.

In contrast, the results from Sved et al., using self-administration of nicotine as the behavioral paradigm, were consistent with the suggestion that MAO inhibition potentiates self-administration of nicotine, but only at doses of nicotine lower than those most frequently used experimentally. In a rat model of schizophrenia this effect of MAO inhibition was abolished. This suggests that, in schizophrenia, the observed high levels of smoking may not be related to MAO inhibition. It also makes clear that different populations of people may respond differently to the MAO inhibition induced by smoking. The evidence for an effect of MAO inhibition within the nicotine self-administration experimental paradigm highlights the need for improved understanding of monoamine oxidase inhibition in smokers. Very low nicotine cigarettes also contain MAO inhibitors in amounts similar to those of ordinary cigarettes. Thus if low nicotine cigarettes become part of attempts to curb smoking rates, tobacco regulators may need to take these additional pharmacological factors into account.

Both of these papers support the idea that factors in tobacco smoke other than nicotine affect behavior. Monoamine oxidase inhibition in tobacco smoke remains the prime candidate to mediate this effect, but much about the effects of MAO inhibition, in humans and experimentally, remains unclear. It seems likely that interplay of effects of MAO inhibition on both the reinforcing and aversive effects of nicotine will eventually explain some of the apparent anomalies in experimental results.

The rate of smoking is very much higher in those with mental health problems. The paper from Taylor et al., concentrated on the evidence relating to smoking dependence in those with attention deficit hyperactivity disorder (ADHD). This review of relevant literature covers the inter-relationship of smoking and tobacco dependence with ADHD. Much of the evidence pertaining to this examines the possibility that people with ADHD may self-medicate by smoking, along with the hypothesis that nicotine use might be beneficial in moderating ADHD symptoms. The results were equivocal. The authors suggest, based on likely dopamine-related biochemical mechanisms, that examining the contribution of MAO inhibition to behavioral modification within ADHD is worthy of serious investigation.

The final paper contributed by Berlowitz et al. concerned the potential for ß-carbolines such as harmane and norharmane, two monoamine oxidase inhibitors present in tobacco, to have pharmacological effects. They summarize the evidence both that monoamine oxidase enzymes are inhibited in smokers, and toward answering the question as to whether these inhibitors are present in physiologically and behaviorally relevant amounts. The authors drew on evidence with respect to smoking and from the use of ahayuasca, where the ß-carboline content of ahayuasca herbal mixtures is believed to potentiate the effect of the hallucinogen N,N-dimethyltryptamine.

In summary, the possibility that the monoamine oxidase inhibitors in tobacco smoke enhance tobacco dependence remains both increasingly likely and, as yet, unproven. Further, the interaction of such MAO inhibition with a variety of different metal health conditions is a puzzle yet to be solved. Given the world-wide public health burden of tobacco smoking on societies, families and individuals, developing our understanding of MAO inhibition in tobacco use has the potential for significant public health impact.

## Author contributions

The author confirms being the sole contributor of this work and has approved it for publication.

## Conflict of interest

The author declares that the research was conducted in the absence of any commercial or financial relationships that could be construed as a potential conflict of interest.

## Publisher's note

All claims expressed in this article are solely those of the authors and do not necessarily represent those of their affiliated organizations, or those of the publisher, the editors and the reviewers. Any product that may be evaluated in this article, or claim that may be made by its manufacturer, is not guaranteed or endorsed by the publisher.

